# Multi-omics integration identifies NK cell-mediated cytotoxicity as a therapeutic target in systemic lupus erythematosus

**DOI:** 10.3389/fimmu.2025.1580540

**Published:** 2025-05-13

**Authors:** Jingjing Zhang, Ling Ma, Hanyin Deng, Wenqian Yi, Alim Tohtihan, Xiaojun Tang, Xiudi Wu, Xuebing Feng

**Affiliations:** ^1^ Department of Rheumatology and Immunology, Nanjing Drum Tower Hospital, Affiliated Hospital of Medical School, Nanjing University, Nanjing, China; ^2^ Department of Rheumatology and Immunology, Nanjing Drum Tower Hospital Clinical College of Nanjing University of Chinese Medicine, Nanjing, Jiangsu, China; ^3^ Department of Rheumatology, The First Affiliated Hospital of Ningbo University, Ningbo, Zhejiang, China

**Keywords:** systemic lupus erythematosus, ATAC-seq, RNA-Seq, NK cells, open chromatin

## Abstract

**Background:**

Systemic lupus erythematosus (SLE) is an autoimmune condition that impacts various organs. Given the intricate clinical progression of SLE, it is imperative to explore novel avenues for precise diagnosis and treatment.

**Methods:**

Peripheral blood mononuclear cells (PBMC) were isolated from 6 SLE patients before and after treatment, 7 healthy controls and 7 disease controls. Assay for Transposase Accessible Chromatin with high throughput Sequencing (ATAC-seq) was used to analyze the chromatin accessibility signatures and RNA-seq was used to identify the differentially expressed genes, mRNA, lncRNA, circRNA, miRNA. Then ATAC-seq and RNA-seq were integrated to further analyze hub genes and pathways. Finally, we validated gene expression levels and examined changes in key genes after treatment through *in vitro* experiments.

**Results:**

Our analysis reveals dynamic changes in chromatin accessibility during the course of disease progression in SLE. Significantly higher numbers of differentially accessible regions, transcripts, genes, mRNA, lncRNA, circRNA, and miRNA were observed in SLE patients compared to other cohorts, with these variances markedly reduced post-treatment. Two gene clusters associated with SLE disease improvement were identified, with a total of 140 genes intersecting with ATAC results. Pathway analysis revealed that NK cell mediated cytotoxicity was the most differentiated and therapeutically altered pathway in SLE patients. Independent sample validation confirmed that the gene expression of this pathway was reduced in SLE patients and associated with disease activity, whereas hydroxychloroquine (HCQ) effectively elevated their expression *in vitro*.

**Conclusion:**

Our findings suggest that these NK cell signature genes may be associated with the complex pathogenesis of SLE. The restoration of NK cell-mediated cytotoxicity may serve as a useful marker of improvement following SLE treatment.

## Graphical Abstract

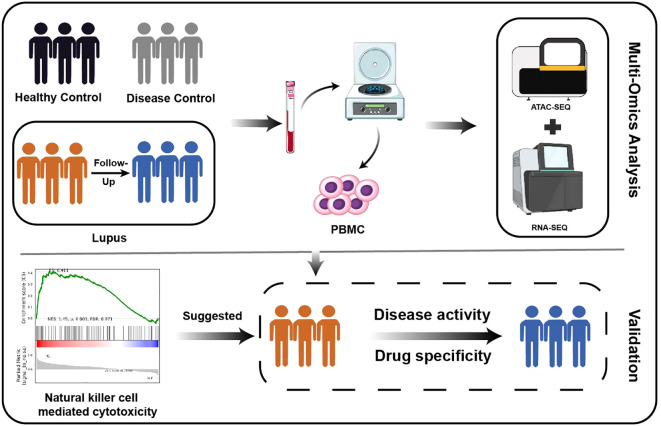

## 1 Introduction

Systemic lupus erythematosus (SLE), a complex autoimmune disease character-ized by diverse clinical manifestations and multi-organ involvement, poses significant challenges to effective patient management. Despite advancements in treatment, a substantial proportion of SLE patients, particularly those with high disease activity, continue to face unfavorable prognoses, and long-term medication often results in detrimental side effects (1, 2) These clinical limitations underscore the urgent need for innovative diagnostic and therapeutic strategies. Given the heterogeneous nature of SLE and the elusive nature of its clinical progression (3), personalized approaches based on precision medicine technologies, notably transcriptomics, hold great promise for improving disease management.

While several studies have identified distinctive transcriptome signatures in SLE patients that can differentiate them from healthy individuals (4), as well as reflecting clinical variations in disease severity and antibody profiles (5, 6), our understanding of the dynamic changes in gene expression throughout the course of SLE, especially in response to treatment, remains incomplete. Epigenetic modifications, operating independently of DNA sequences, play a crucial role in regulating gene ex-pression, including DNA methylation, histone modifications, chromatin accessibility, and non-coding RNAs (7). Of these, chromatin accessibility, which governs the binding of regulatory elements and transcription factors, is particularly pivotal in transcriptional control (8). The assay for Transposase Accessible Chromatin using sequencing (ATAC-seq) provides a straightforward and scalable approach to explore open chromatin regions.

When integrated with RNA-seq, ATAC-seq helps identify regulatory changes that impact gene expression crucial for understanding disease’s pathogenesis. There have been several successful applications of combining ATAC-seq with RNA-seq, such as the discovery of candidate genes and transcriptional factors associated with hemangiomas (9). These findings offer new perspectives on understanding the pathogenesis of complex diseases. There have been attempts to explore the pathogenesis of SLE in conjunction with the application of ATAC-seq combined with transcriptome analysis (10, 11), but to date no dynamic changes in these multi-omics before and after treatment have been reported. In this study, we conducted integrated analysis of ATAC-seq and RNA-seq to explain the epigenetic and transcriptional landscape of SLE, focusing on gene clusters that underwent changes after treatment. The research results may provide new references for the diagnosis and treatment of SLE.

## 2 Materials and methods

### 2.1 Sample preparation

The study protocol was approved by the Ethics Committee of the Affiliated Drum Tower Hospital of Nanjing University Medical School (No. 202218801), and written informed consent was obtained from all participants. A 10-ml peripheral blood sample was collected from six patients with SLE, seven patients with rheumatoid arthritis (RA), and seven healthy controls (HC) at the Affiliated Drum Tower Hospital of Nanjing University Medical School and Ningbo First Hospital, and then peripheral blood mononuclear cells (PBMCs) were isolated by the Ficoll-Hypaque discontinuous gradient method and preserved at -80°C until assayed. Patients with SLE and RA met the 1997 American College of Rheumatology (ACR) classification criteria and the 1987 ACR criteria (12, 13). Disease activity in SLE patients was assessed by the Systemic Lupus Erythematosus Disease Activity Index (SLEDAI-2K) (14), and all SLEDAI scores of the included SLE patients were greater than 10. In addition, patients with SLE were followed up for an average of 5.3 months and peripheral blood was collected again at their next hospital visit as the post-treatment control. The main characteristics of each group were shown in **Supplementary Table S1**. To validate the results of the multiomics study, another 16 SLE patients and 16 age, gender matched HC were recruited from the Affiliated Drum Tower Hospital of Nanjing University Medical School, and their information was summarized in **Supplementary Table S2**.

### 2.2 ATAC sequencing

Cell activity was assessed with a Trypan blue assay and then ATAC-seq was performed as described (15). Briefly, nuclei were extracted from samples, and the nuclei pellet was resuspended in the Tn5 transposase reaction mix. After transposition, PCR was performed to amplify the library by adding two different barcodes. Following the PCR reaction, libraries were purified with the AMPure beads and sequenced on an Illumina Hiseq platform.

The reads were aligned to hg38 using BWA (v0.7.12) with standard parameters. MACS2 (v2.1) was used for peak calling with the parameters ‘-q 0.05 –call-summits –nomodel –shift -100 –extsize 200 –keep-dup all’. Peak simulations per input read were performed using aligned and de-duplicated BAM files without any additional filtering. Peaks of different groups were merged using bedtools. Differential analysis of accessible regions was performed using the R package DESeq2(v1.36.0). Peaks with an absolute log2 fold change greater than 1 (|log2FC|>1) and p-value < 0.05 were considered differentially accessible. ChIPseeker (16)(v1.24.0) was used to annotate the differential peaks. Motif enrichment was calculated using HOMER (default parameters) on DE(Differential expression) peaks (17). The Integrative Genomics Viewer (IGV 2.19.3) was used for data visualization.

### 2.3 RNA sequencing

Total RNA was extracted from PBMCs using Trizol reagent (Vazyme). RNA quality control was performed using NanoPhotometer and Agilent 2100 RNA Nano 6000 Assay Kit (AgilentTechnologies, CA, USA). Each sample was prepared using a total of 5 μg RNA. The lncRNA, mRNA and circRNA sequencing libraries were generated by TruSeq^®^ RNA Sample Prep Kit (Illumina, USA) following manufacturer’s recommendations. For the small RNA library, Sequencing libraries were generated using NEBNext^®^Multiplex Small RNA Library Prep Set (NEB, USA). The library fragments were purified with AMPure XP system. The generated libraries were then submitted for Illumina sequencing.

Raw data of FASTQ format were filtered to obtain clean data with high quality. Clean reads for each sample were mapped to hg38 with the software HISAT2 and Bowtie. The circRNA were detected and identified using find_circ (18) and CIRI2 (19). Mapped small RNA tags were used to looking for known miRNA and miREvo (20) and miRDeep2 were integrated to predict novel miRNA. The target genes of miRNAs were predicted by miRanda.The miRNA expression levels were estimated by TPM (transcript per million). Differential expression analysis of two groups was performed using the DESeq2 R package (v1.36.0). Differentially expressed genes (DEGs) were defined as those with |log2 fold change| ≥1 and p-value < 0.05.

### 2.4 Functional and cluster analysis

Gene Ontology (GO) analysis and Kyoto Encyclopedia of Genes and Genomes (KEGG) analysis were performed using the ‘clusterProfiler’ R package (21) or the DAVID Database (v2021). The Mfuzz was used for sub-clustering models. Principal component analysis (PCA) were applied to evaluate the signature of transcriptomics. Receiver operating characteristic (ROC) was conducted by Graphpad Prism (v9.0) and calculated the corresponding area under the curve (AUC).

### 2.5 ceRNA network construction

According to the ceRNA (endogenous competitive RNAs) hypothesis, lncRNAs can act as miRNA sponges, competing for microRNA binding to regulate mRNA activity (22). The miRNA-lncRNA regulatory relationship was predicted using starBase (v2.0). The miRNA-circRNA and miRNA-gene regulatory network was predicted using miRanda. Cytoscape was used to construct a lncRNA-miRNA-mRNA and circRNA-miRNA-mRNA networks.

### 2.6 Immune infiltration analysis

All samples were analyzed for immune infiltration using the CIBERSORT algorithm (https://cibersort.stanford.edu/). Briefly, based on a reference set of 22 known immune cell subtypes, the content of each immune cell subset was calculated using a deconvolution method.

### 2.7 GEO database analysis

Gene expression profiles with series number GSE211700 were downloaded from the Gene Expression Omnibus (GEO) database (http://www.ncbi.nlm.nih.gov/geo/).The total sample of the two patient groups is 30 and the healthy controls are 10 subjects. Limma of R/bio-conductor was used for screening of the DEGs.

### 2.8 Quantitative real-time PCR

The extracted RNA was reverse transcribed using HIScrip III qRT SuperMix (R323, Vazyme). Each cDNA sample was amplified and quantified by real-time PCR using a Taq-pro Universal SYBR Q-PCR Master Mix (Q712, Vazyme). The relative gene expression levels were calculated by using the 2^−△△Ct^ method, with normalization to glyceral-dehyde-3-phosphate dehydrogenase (GAPDH). The primers used in this study were detailed in **Supplementary Table S3**.

### 2.9 Cell cultures

PBMCs were collected from SLE patients and resuspended in RPMI 1640 medium supplemented with 10% FBS and 1% penicillin-streptomycin. Cells were grown in 96-well plates at 37°C in 5% CO_2_ and treated with 1um prednisone (MedChemExpress, USA), 40um cyclophosphamide (MedChemExpress, USA) and 20um hydroxychloroquine (MedChemExpress, USA) respectively as described before for 24 hours (23, 24).

### 2.10 Statistical analysis

Statistical analyses were performed using R (v4.2.2) and Graphpad Prism (v9.0). Student’s t-test was used to test the significance of the difference between two groups. Relations between two variables were evaluated using Spearman correlation test. A two-tailed p-value < 0.05 was considered statistically significant.

## 3 Results

### 3.1 ATAC-seq revealed massively differentially accessible regions in SLE patients

**Figure 1:**
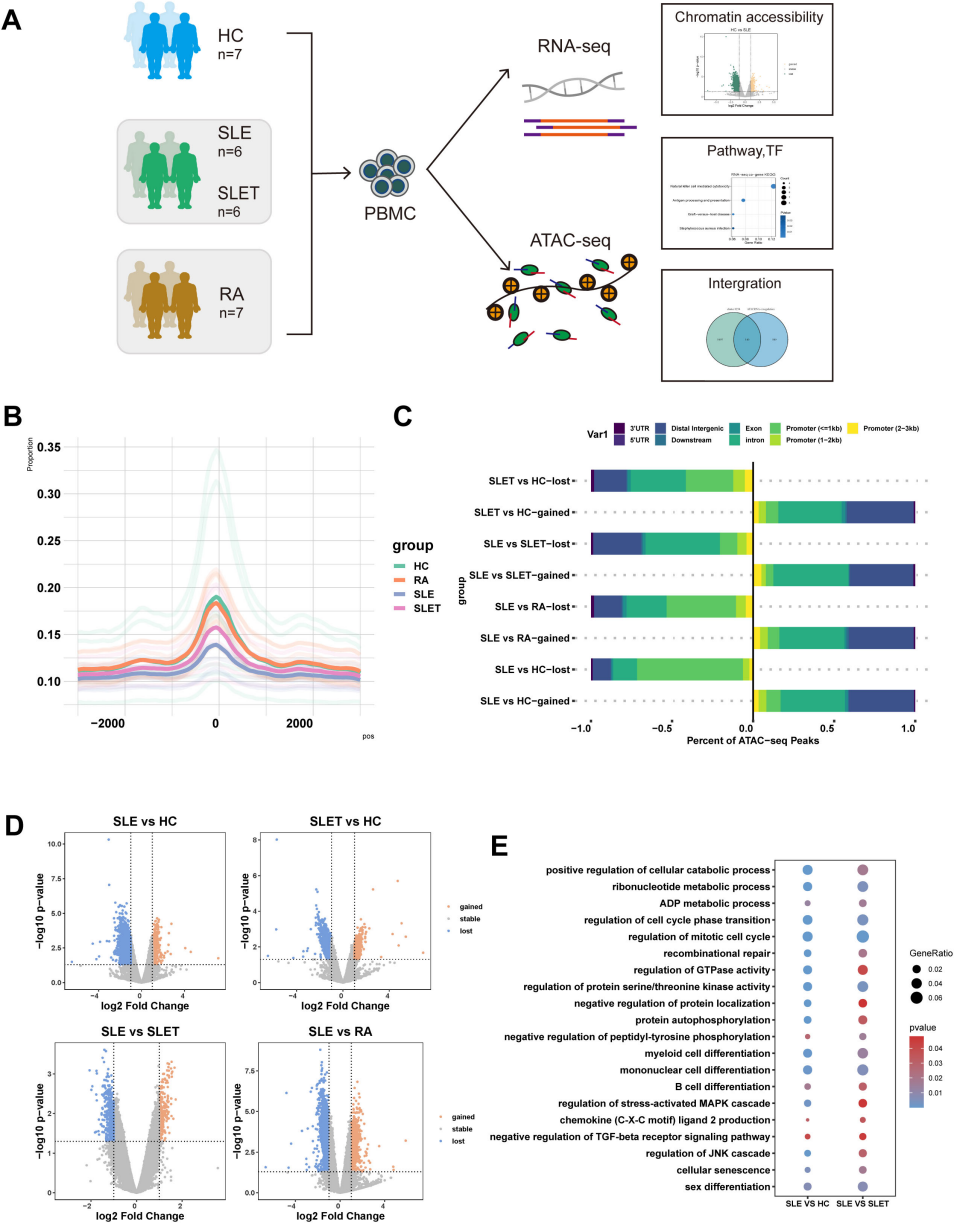
SLE chromatin accessibility signatures. **(A)** The flowchart of the research design (HC: healthy control, SLE: patients with systemic lupus erythematosus, SLET: same SLE patients after treatment, RA: patients with rheumatoid arthritis). **(B)** Histogram of the average signal distribution on the 3kb upstream and downstream regions of transcription start sites (TSS) in each sample group. **(C)** Proportions of cis-acting elements distribution in the regions corresponding to the DA peaks of each group. **(D)** Volcano plots of differentially accessible chromatin regions (DARs) among each group (|log2FC|> 1 and P-value<0.05). **(E)** Common pathways for GO analysis of DARs between SLE vs HC and SLE vs SLET.

PBMCs before and after treatment were collected from 6 SLE patients, tested for both ATAC-seq and RNA-seq, with samples of HC and RA patients as controls (**Figure 1A**). As expected, the ATAC-seq signal was predominantly enriched at the transcription start site (TSS). The openness at TSS region was lower in SLE patients compared with HC and RA patients, but markedly increased after treatment (**Figure 1B**). Most of the lost peaks between SLE vs HC were enriched in promoter regions. After treatment, a majority of lost peaks were enriched in introns and intergenic regions, suggesting the possible presence of enhancers and silencers (**Figure 1C**). Comparative analysis of differentially accessible regions (DARs) revealed striking chromatin remodeling patterns associated with disease status and treatment response (**Figure 1D**). Notably, the SLE vs HC comparison demonstrated the most pronounced differences, with 1,338 gained peaks (0.88%) and 11,778 lost peaks (7.70%). However, treatment-mediated attenuation was observed in SLET patients, where comparisons with HC showed a reduction to 816 gained peaks (0.56%) and 2,201 lost peaks (1.51%). Moreover, SLE vs SLET displayed 256 gained (0.14%) and 473 lost peaks (0.27%), while SLE vs RA comparisons showed 2,087 gained (1.16%) and 5,444 lost peaks (3.03%). **Supplementary Table S5** showed genes corresponding to DARs that are specifically altered in SLE but not in SLET.

Through functional enrichment analysis, significant enrichment of the pathways such as mononuclear cell differentiation, regulation of protein serine/threonine kinase activity and regulation of mitotic cell cycles were identified in both SLE vs. HC and SLE pre- and post-treatment comparisons (**Figure 1E**). Our data revealed that there were many different chromatin open regions in SLE patients. Meanwhile, chromatin open regions captured by ATAC-seq were often binding sites for transcription factors (TF). As shown in **Supplementary Table S4**, forkhead family members were mainly enriched in the gained-DARs, while ETS family members were mainly enriched in both SLE vs. HC and SLE pre- and post-treatment comparisons.

### 3.2 Transcriptome analysis indicated extensive differential transcripts in SLE patients

**Figure 2:**
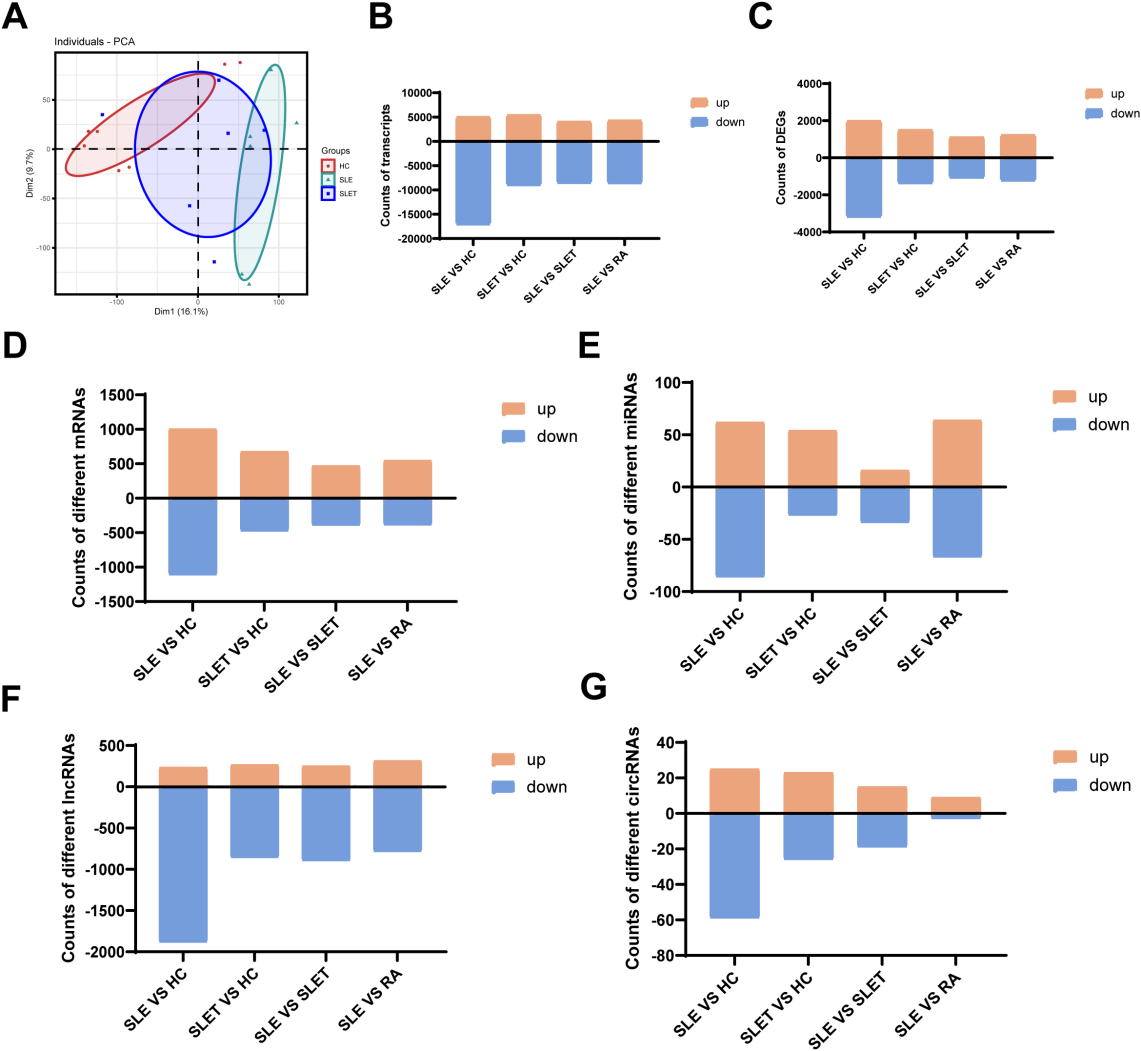
Landscape of transcriptome in SLE. **(A)** Principal component analysis (PCA) of transcriptome. **(B)** The number of different transcripts between different groups. **(C)** The number of DEGs between different groups. **(D)** The number of different mRNAs between different groups. **(E)** The number of different miRNAs between different groups **(F)** The number of different lncRNAs between different groups. **(G)**The number of different circRNAs between different groups.

The whole transcriptome sequencing results were analyzed using PCA. The PCA revealed that the transcriptional profiles of SLE patients significantly differed from HC. However, after treatment, the transcriptional profiles of SLE patients showed a tendency to converge toward those of HC (**Figure 2A**). To visualize the distribution of this disparity, the distribution of differentially expressed transcripts and other tested parameters was compared across groups. Our results showed that the highest number of total differential transcripts, differential genes, differential mRNAs, differential miRNAs, differential lncRNAs, and differential circRNAs were all found between SLE vs. HC, especially those with down-regulated expression (**Figures 2B–G**). Similar to the results of ATAC-seq, this profile improved significantly after treatment in SLE patients.

### 3.3 Identification of DEGs associated with SLE treatment

**Figure 3:**
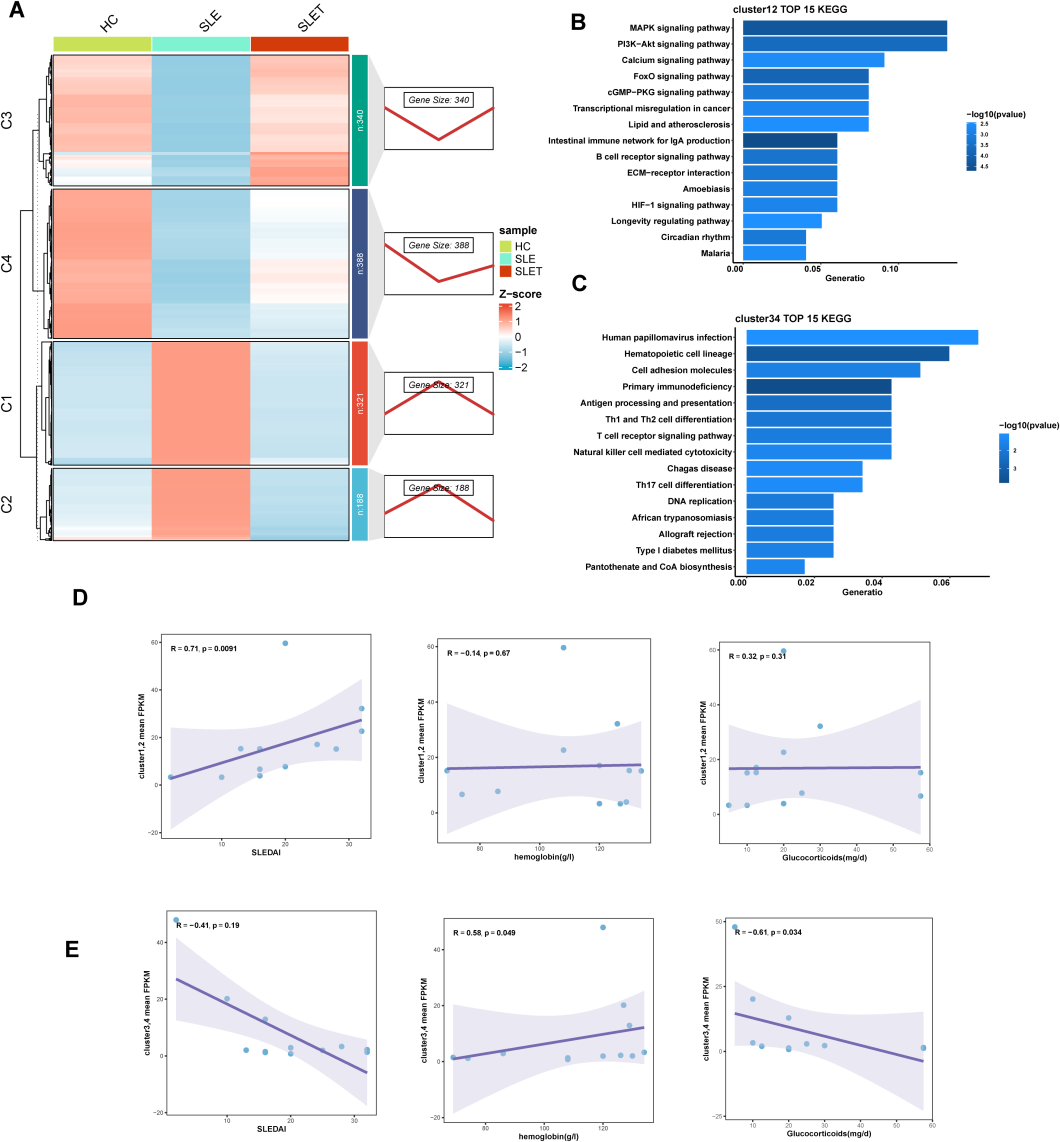
Different gene expression patterns related to SLE treatment. **(A)** The right panel displays expression patterns obtained from Mfuzz clustering. The left panel shows the clustering heatmap. **(B, C)** The TOP 15 KEGG enrichment analysis results of cluster1-2 **(D)** and cluster3-4 **(E)**. **(D, E)** Scatter plot of correlation between average FPKM values and SLEDAI, hemoglobin, hormone dosage for genes in cluster1-2 **(D)** and cluster3-4 **(E)**.

To identify gene clusters that underwent changes after SLE treatment, DEGs from HC as well as pre- and post-SLE treatment groups were clustered and analyzed. As shown in **Figure 3A**, all DEGs were clustered into two groups: cluster1-2 (C1, C2) and cluster3-4 (C3, C4), which consisted of 509 and 728 DEGs, respectively. Gene expression in cluster1-2 was significantly higher in SLE patients than in HC and decreased or trended down after treatment, while that in cluster3-4 was the opposite. KEGG analysis revealed that cluster1-2 genes were primarily involved in MAPK signaling pathway, PI3K-Akt signaling pathway and B cell receptor signaling pathway (**Figure 3B**), while cluster3-4 genes were enriched in Th1 and Th2 cell differentiation, T cell receptor signaling pathway and natural killer cell mediated cytotoxicity (**Figure 3C**). Next, the mean standardized expression levels of all genes in the gene clusters were correlated with disease activities, hemoglobin levels and glucocorticoid dosages to understand the clinical significance of these gene clusters. The results showed that the mean expression levels of genes in clusters 1-2 were significantly positively correlated with SLEDAI scores (**Figure 3D**), while the mean expression levels of genes in clusters 3-4 were negatively correlated with glucocorticoid doses (**Figure 3E**). Thus, gene expression alteration in clusters 1-2 may primarily reflect changes in disease activity.

### 3.4 Identification of miRNAs associated with SLE treatment

**Figure 4:**
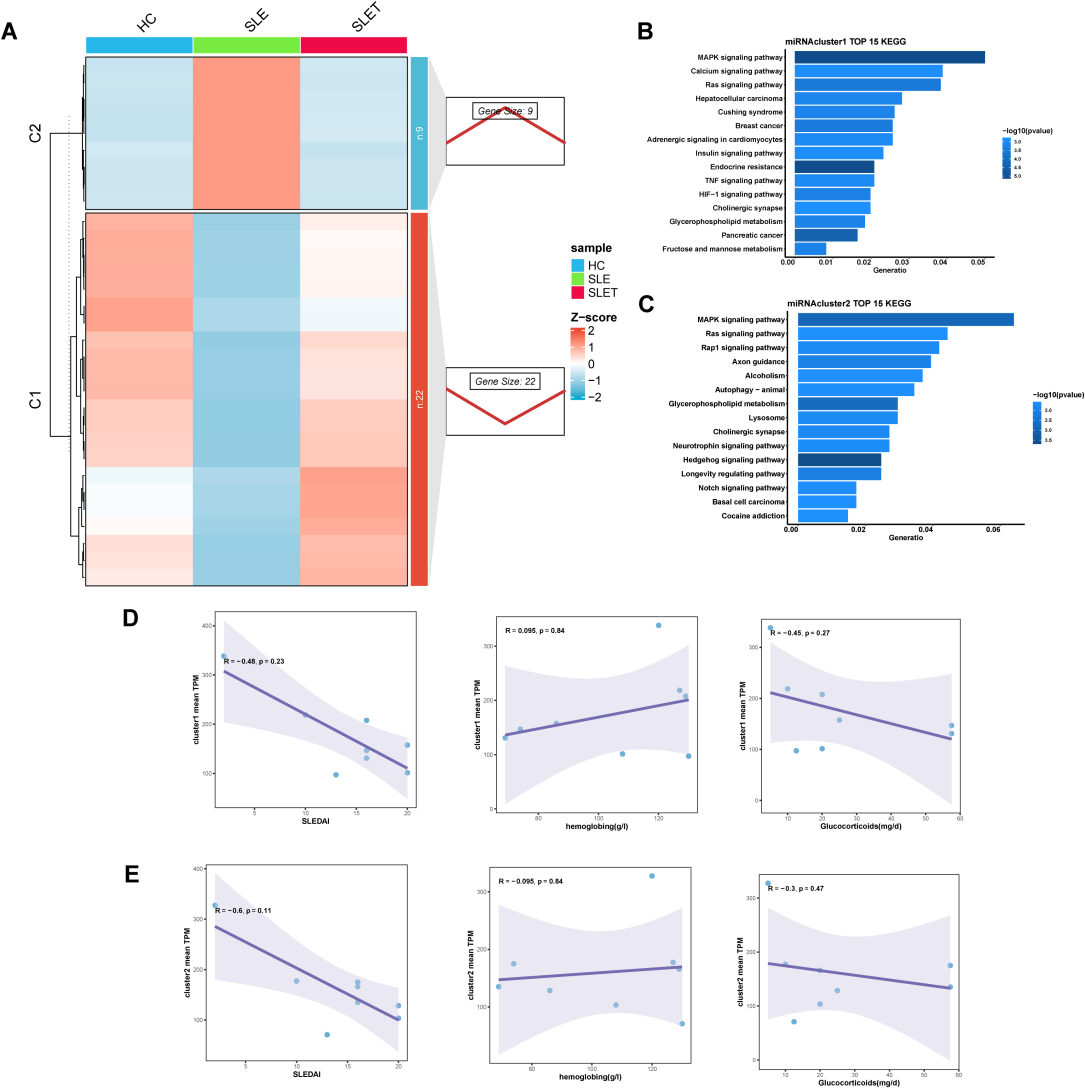
Different miRNA expression patterns related to SLE treatment. **(A)** The right panel displays miRNA expression patterns obtained from Mfuzz clustering. The left panel shows the clustering heatmap. **(B, C)** The TOP 15 KEGG enrichment analysis results of cluster1 **(D)** and cluster2 **(E)**. **(D, E)** Scatter plot of correlation between average TPM values and SLEDAI, hemoglobin, hormone dosage for genes in cluster1 **(D)** and cluster 2 **(E)**.

Similarly, the differential miRNAs before and after SLE treatment could also be categorized into two clusters by cluster analysis: cluster 1 had 22 miRNAs and cluster 2 had 9 miRNAs (**Figure 4A**). Enrichment analysis of the genes targeted by miRNA showed that cluster 1 was associated with MAPK signaling pathway, Calcium signaling pathway, and Ras signaling pathway (**Figure 4B**). Cluster 2 was associated with MAPK signaling pathway, Ras signaling pathway, and Rap1 signaling pathway (**Figure 4C**). However, cluster 1 and 2 showed no significant correlation with SLEDAI scores, glucocorticoid dosages, and hemoglobin levels (**Figures 4D, E**), which was likely related to the small sample size for miRNA sequencing.

Then we used these 31 key miRNAs to search databases for over 5,000 possible target genes. We combined these with genes that also changed after SLE treatment, constructing miRNA-gene interaction pairs related to SLE treatment. By overlapping the differentially expressed circRNAs and lncRNAs from SLE vs HC and SLET vs HC comparisons, we identified hub-circRNAs and hub-lncRNAs that were altered in SLE but remained unchanged in SLET(**Supplementary Figures S1A, B**). We then predicted the target miRNAs of these hub-circRNAs and hub-lncRNAs, integrating them with the previously identified miRNA-gene pairs to construct lncRNA-miRNA-mRNA and circRNA-miRNA-mRNA networks (**Supplementary Figures S1C, D**). Among them, has-miR-486-3p has the highest frequency in both networks.

### 3.5 Integrated analysis suggested an essential role for NK cell-mediated cytotoxicity pathway in lupus

**Figure 5:**
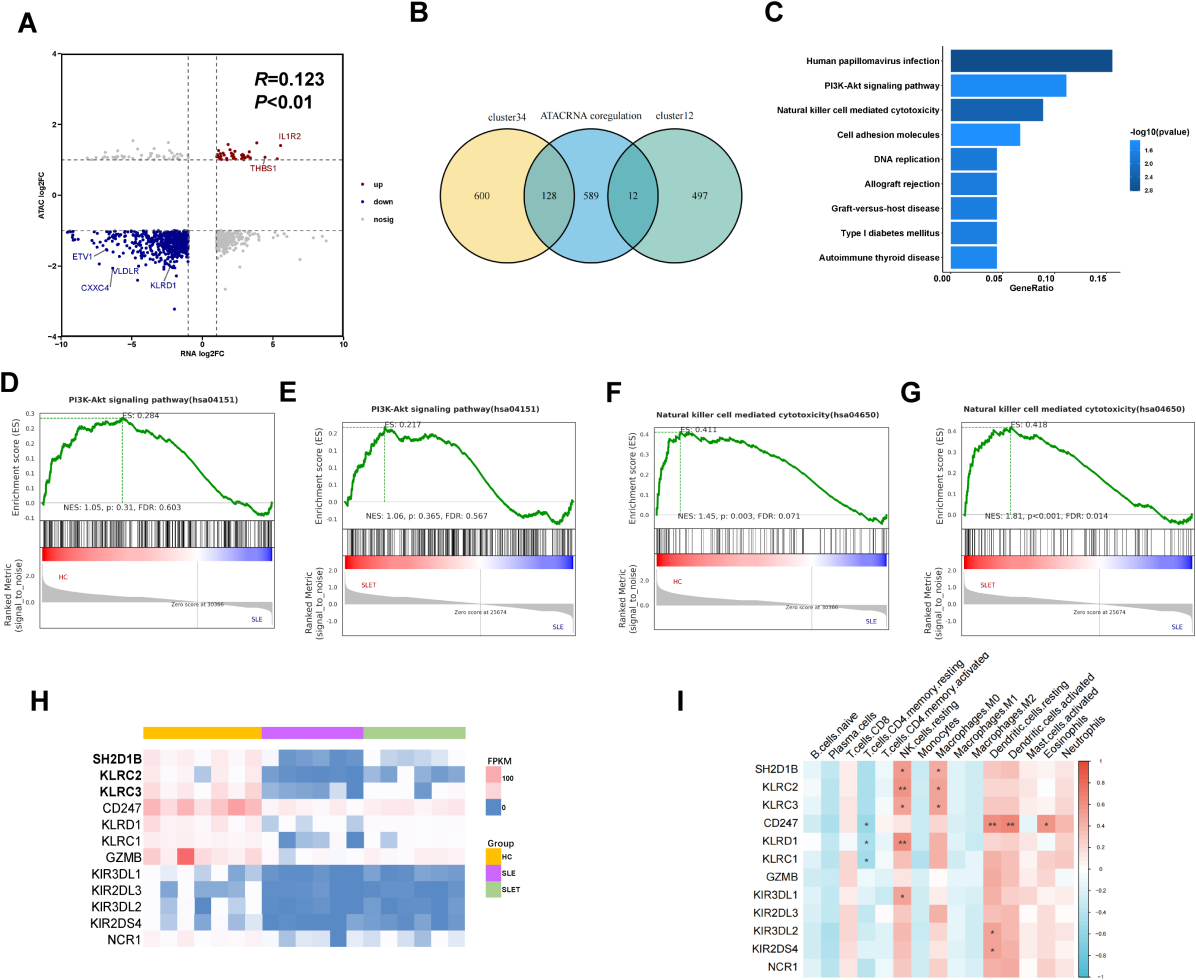
Integrated analysis of ATAC-seq and RNA-seq. **(A)** The nine-quadrant plot showing relevant changes of chromatin accessibility and gene differential expression **(B)** Venn diagram showing the amount of overlap genes between cluster1-4 and ATAC-RNA coregulation. **(C)** TOP 9 KEGG enriched pathways of 140 genes. **(D, F)** GSEA enrichment analysis in SLE vs HC. **(E, G)** GSEA enrichment analysis in SLE pre- and post-treatment. **(H)** The heatmap showing the expression level of 12 genes in pathway of NK cell mediated cytotoxicity. **(I)** Correlation between 12 gene expressions and various immune cells. Data are presented as mean ± SD. **P ≤ 0.01, *P ≤ 0.05.

Combined analysis of ATAC-seq and RNA-seq showed a positive correlation between the two tests (**Figure 5A**), confirming that increased chromosome accessibility is associated with higher gene transcription. The genes with consistent changes were integrated with the cluster1-4 genes, and a total of 140 genes were found to be intersected (**Figure 5B**). **Supplementary Table S6** shows the expression levels and localization information of these genes. Moreover, we also constructed ceRNA networks, resulting in interaction relationships of circRNA-miRNA-genes and lncRNA-miRNA-genes (**Supplementary Figures S2A, B**).

Pathway enrichment analysis was performed using the KEGG database to assess the functional roles of the 140 integrated genes. The analysis revealed a significant enrichment of these genes in pathways related to PI3K-Akt signaling and NK cell-mediated cytotoxicity, implicating their involvement in immune surveillance mechanisms that are potentially dysregulated in SLE (**Figure 5C**). However, GSEA analysis showed that the NK cell-mediated cytotoxicity pathway, but not PI3K-Akt signaling pathway, was differential in the comparison of SLE patients with HC and SLE patients after treatment (**Figures 5D–G**). As shown in the heatmap, the expression of genes in NK cell-mediated cytotoxicity pathway was markedly downregulated in SLE patients and showed a rebound trend after treatment (**Figure 5H**). **Supplementary Figures S3A–D** also showed the DARs signaling detected by ATAC-seq at NK cell receptor genes. Next, we analyzed the expression of the above genes in different immune cells using the CIBERSORT algorithm. As shown in **Figure 5I**, these genes were predominantly highly expressed in NK cells, but were also aberrantly present in CD4^+^ T cells, macrophages, and dendritic cells.

### 3.6 Validation of gene expression of the NK cell-mediated cytotoxicity pathway

**Figure 6:**
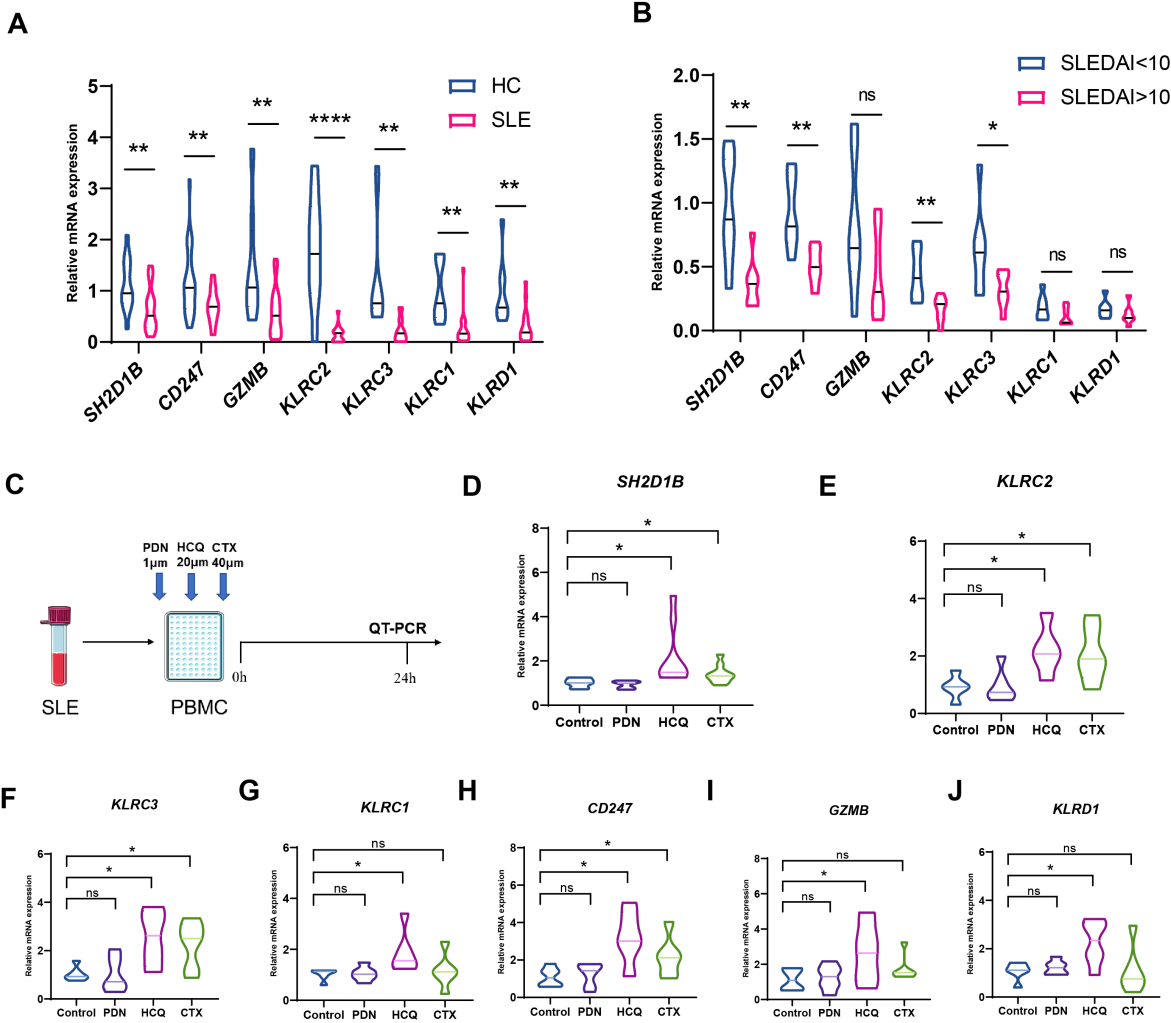
Changes of hub genes after treatment. **(A)** The mRNA expression levels of *SH2D1B, CD247, GZMB, KLRC2, KLRC3, KLRC1* and *KLRD1* were validated by q-PCR **(B)** The mRNA expression levels of *SH2D1B, CD247, GZMB, KLRC2, KLRC3, KLRC1* and *KLRD1* between SLEDAI<10 group and SLEDAI>10 group. **(C)** Schematic diagram of a drug intervention experiment. **(D–J)** The mRNA expression levels of *SH2D1B, CD247, GZMB, KLRC2, KLRC3, KLRC1* and *KLRD1* after co cultivation with different drugs. PDN, prednisone. CTX, cyclophosphamide; HCQ, hydroxychloroquine. ****P ≤ 0.0001, **P ≤ 0.01, *P ≤ 0.05, ns, no significance.

The mRNA expression levels of seven major genes in this pathway were verified by qPCR, and all of them were significantly down-regulated in SLE patients (**Figure 6A**), with *SH2D1B, CD247, KLRC2 and KLRC3* being more pronounced in those with SLEDAI scores higher than 10 (**Figure 6B**). We also validated using a public GEO database, which is highly consistent with our results (**Supplementary Figures S3E, F**). To observe the changes in the expression of these key genes after various medicines, PBMCs of SLE were isolated and co-cultured with prednisone, hydroxychloroquine and cyclophosphamide that commonly used for SLE treatment for 24 hours (**Figure 6C**). To our surprise, hydroxychloroquine treatment significantly increased the expression of these genes, with an even stronger effect than cyclophosphamide (**Figures 6D–J**).

## 4 Discussion

The heterogeneity of SLE patients brings great challenges in the evaluation of this autoimmune disorder. Multiomics technology has emerged as a promising tool that rapidly and efficiently identifies key genes involved in the onset and progression of SLE. This approach offers a novel framework that could revolutionize the diagnosis and treatment strategies for this disease. In this study, we demonstrated a significant reduction in DAR regions after SLE treatment, and also revealed changes in gene clusters and miRNA clusters associated with treatment. By integrating ATAC-seq and RNA-seq, we found that down-regulation of the signaling pathway for NK cell-associated cytotoxicity is a shared basis for disease activity in patients with SLE, whereas up-regulation of gene expression in this pathway after treatment may predict a better outcome for patients. *In vitro* studies showed that hydroxychloroquine was most helpful in restoring the abnormalities of this pathway.

ATAC-seq is a widely used method to detect chromatin accessibility, which requires fewer cells than other epigenetic analysis. Epigenetic changes can reflect the dynamic regulation process of genes. In this study, we observed significant differences in DARs between HCs and SLE. Importantly, these accessibility patterns altered following treatment, which were mainly related to immune metabolism and inflammation. Many studies have confirmed that the chromatin accessibility is related to the pathogenesis of SLE in various cells type. For example, in naïve B cells from SLE biobanks, chromatin accessibility around the activation genes was altered, which was correlated with the accessibility change of BATF, a TF binding motif (25). In addition, research on CD4+T cells from SLE demonstrated that the chromatin accessibility of CD4+T cells was related to the clinical severity of SLE (11), and the accessible area of CD4+T cells was related to T helper 17 cell differentiation and cell cycle (10). However, it should be noted that lots of DARs did not mapped to DEGs, indicating that chromatin accessibility has limited ability to regulate genes, and there may be multiple other regulatory regions (10).

Chromatin accessibility is closely related to the binding of regulatory elements or transcription factors, which is particularly important for gene expression regulation. ATAC-seq has been used to determine the mechanism of gene expression regulation. In this study, we performed motif analysis of DARs and found that they were mainly related to the regulation of ETS and FOX transcription factors. A genome-wide study in Asian populations found that ETS1 is associated with susceptibility to SLE (26). In addition, ETS1-deficient mice exhibit lupus-like symptoms, mainly characterized by high titers of autoantibodies and deposition of immune complexes in the kidneys (27). Although the involvement of ETS in the onset of lupus remains unclear, future studies should focus on delineating the specific pathways through which ETS transcription factors may influence SLE pathogenesis, possibly involving inflammatory signaling cascades. FOX transcription factors play an important role in lymphocyte activation and proliferation (28), and increasing evidence suggests that FOX transcription factors are implicated in the pathogenesis of SLE (29, 30).

By comparing the number of differentially elements (DARs, genes, miRNAs, lncRNAs, circRNAs) between different groups, we observed the greatest total number of differences between the SLE and healthy controls, suggesting widespread epigenetic and transcriptomic dysregulation in SLE patients. Notably, the number of these differentially elements decreased significantly after treatment, indicating that the treatment may have partially reversed these abnormalities. However, careful consideration is needed when interpreting the numbers of differentially elements observed between the groups. As a whole-genome differential analysis of monozygotic twins revealed that approximately 25% of allele-specific expression differences stem from differences in genetic background (31), the number of differentially elements observed between groups may be influenced by the potential impact of baseline genetic heterogeneity between individuals. This impact may add complexity to the interpretation of the results, especially in SLE, which have complex genetic underpinnings.

By integrating ATAC-seq and RNA-seq data, we found genes related to NK cell mediated cytotoxicity pathways were significantly altered in the disease group. The pathogenesis of SLE is mainly related to B cell producing autoantibodies and T cells, while the role of NK cells in the pathogenesis of SLE is still controversial (32). Past studies have shown that the proportion and absolute number of NK cells in SLE patients are significantly reduced, and their cytotoxicity is reduced (33, 34). However, many studies have found enhanced NK cytotoxicity in SLE. For example, one research reported that down-regulation of CD3ζ in SLE patients increased the natural cytotoxicity of NK cells (35). Another study found that the activation of NK cells in the kidneys of lupus mice produced cytotoxic substances and pro-inflammatory cytokines, which induced tissue damage (36).While the role of NK cells remains under debate, our study reveals that other cell subsets are also involved in the regulation of disease activity, further complicating the immune landscape in SLE. Similar to the inability of GrB Bregs to inhibit the inflammatory response of Th1, Th2, and Th17 cells as reported in the literature (37), the impairment of NK cell-associated cytotoxicity in SLE may be related to down-regulation of the TCR zeta or a decreased ability to induce T cell apoptosis.

Our study also found that NK cell-mediated cytotoxicity was restored in SLE patients after treatment. Previous studies have reported that high doses of cyclophosphamide may reduce the number and activity of NK cells by inhibiting the formation of NK cell precursors (38). Patients receiving prednisone also seem to have lower natural killer cell activity (39, 40). Hydroxychloroquine is a 4-aminoquinoline derivative antimalarial drug that has complex immunomodulatory effects in SLE. There are currently no reports on its effect on NK cell activity. Hydroxychloroquine has been reported to inhibit autophagy by binding to lysosomes and autophagosomes (41). Autophagy reduces the levels of granzyme B and other enzymes in NK cells under conditions such as hypoxia (42). Our *in vitro* experiments found that the restoration of NK cell-mediated cytotoxicity in lupus patients was mainly dependent on the use of hydroxychloroquine. A possible explanation is that hydroxychloroquine inhibits the inhibitory effect of NK cell autophagy.

Although our study has made some significant discoveries, it is still limited in certain aspects. Firstly, our sample size is relatively small and we may not have obtained comprehensive results during the analysis process, which requires further research on a large-scale cohort. Secondly, we have not elucidated the molecular mechanisms of the identified genes, and more *in vitro* and *in vivo* experimental studies are necessary in the future to comprehensively understand the pathogenic mechanisms of these key genes. Lastly, we conducted sequencing analysis on the overall PBMC population and did not specifically examine the characteristics of individual cell type changes, such as NK cells.

## 5 Conclusion

Our study reveals the dynamics change of chromatin accessibility in SLE patients before and after treatment and identifies pivotal genes associated with SLE treatment. Joint analysis of ATAC-seq and RNA-seq showed that NK cell-mediated cytotoxicity pathways were most characterized by their alterations in SLE patients after treatment. These findings provide new insights and evidence for further research on the underlying pathogenesis of SLE.

## Data Availability

The data presented in the study are deposited in the GEO repository/**Supplementary Material**, the accession number is GSE295130. Further inquiries can be directed to the corresponding author.

## Supplementary material

Supplementary Figure S1 The ceRNA networks. **(A)** The venn diagram of circRNAs between differerent groups. **(B)** The venn diagram of lncRNAs between differerent groups. **(C)** The circRNA-miRNA-gene networks; **(D)** The lncRNA-miRNA-gene networks;

Supplementary Figure S2 The ceRNA networks in 140 overlapped genes. **(A)** The circRNA-miRNA-gene networks; **(B)** The lncRNA-miRNA-gene networks;

Supplementary Figure S3 Genomic localization and RNA-seq expression analysis of NK cell receptor genes. **(A)** IGV shows peaks located around NK cell receptor genes in different group; **(B)** The differential analysis of expression abundance for NK cell receptor genes in RNA-seq data from the public GEO database. ***P ≤ 0.001, **P ≤ 0.01, *P ≤ 0.05.
