# Supplementary figures and images for: Multi-omics integration identifies NK cell-mediated cytotoxicity as a therapeutic target in systemic lupus erythematosus
Source: Front Immunol. 2025 May 13;16:1580540. doi: 10.3389/fimmu.2025.1580540 (PMC12106370; doi:10.3389/fimmu.2025.1580540)

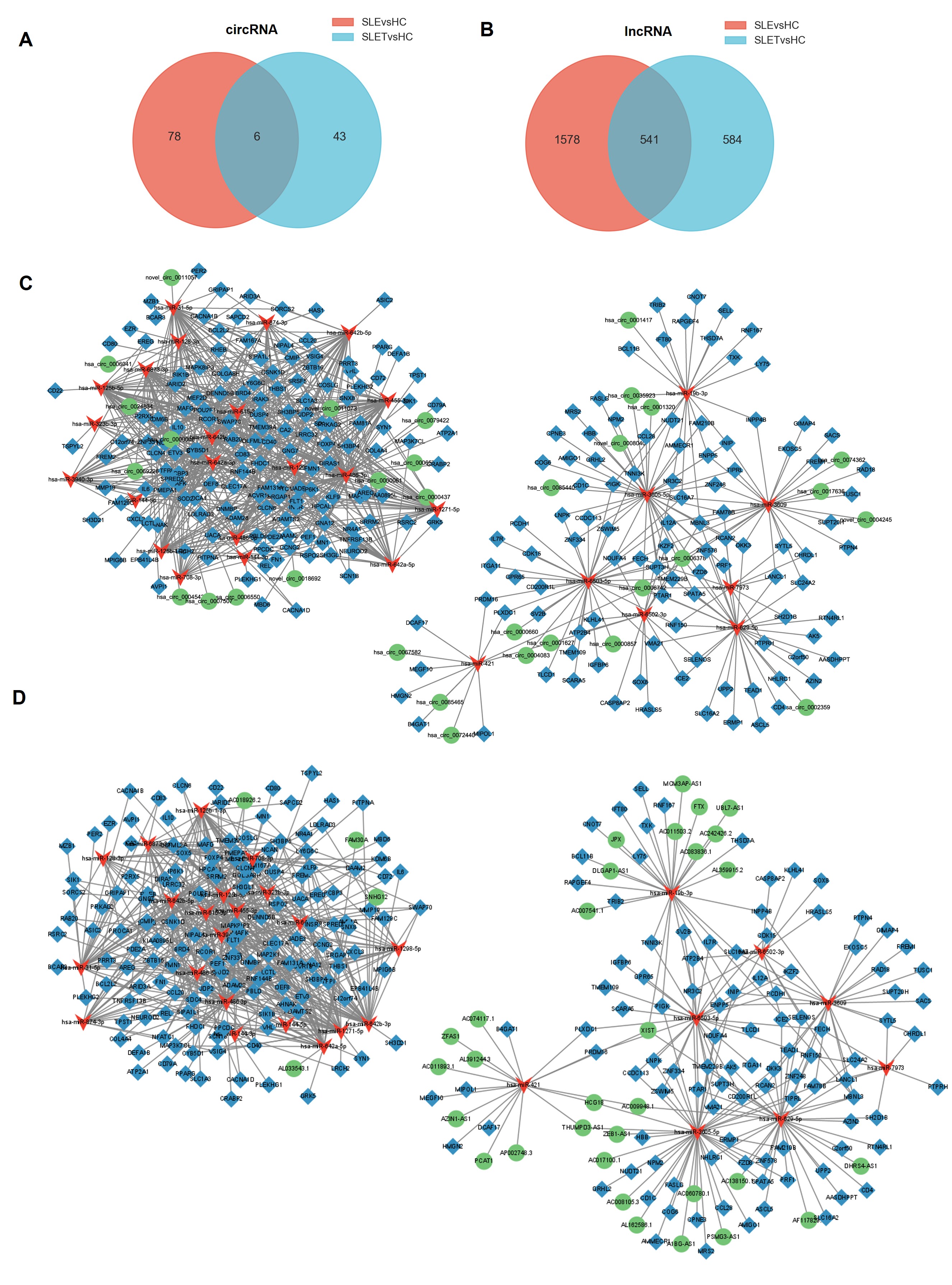

Supplement: Supplementary Figure S1 — The ceRNA networks. (A) The venn diagram of circRNAs between differerent groups. (B) The venn diagram of lncRNAs between differerent groups. (C) The circRNA-miRNA-gene networks; (D) The lncRNA-miRNA-gene networks; [file Image1.jpeg]

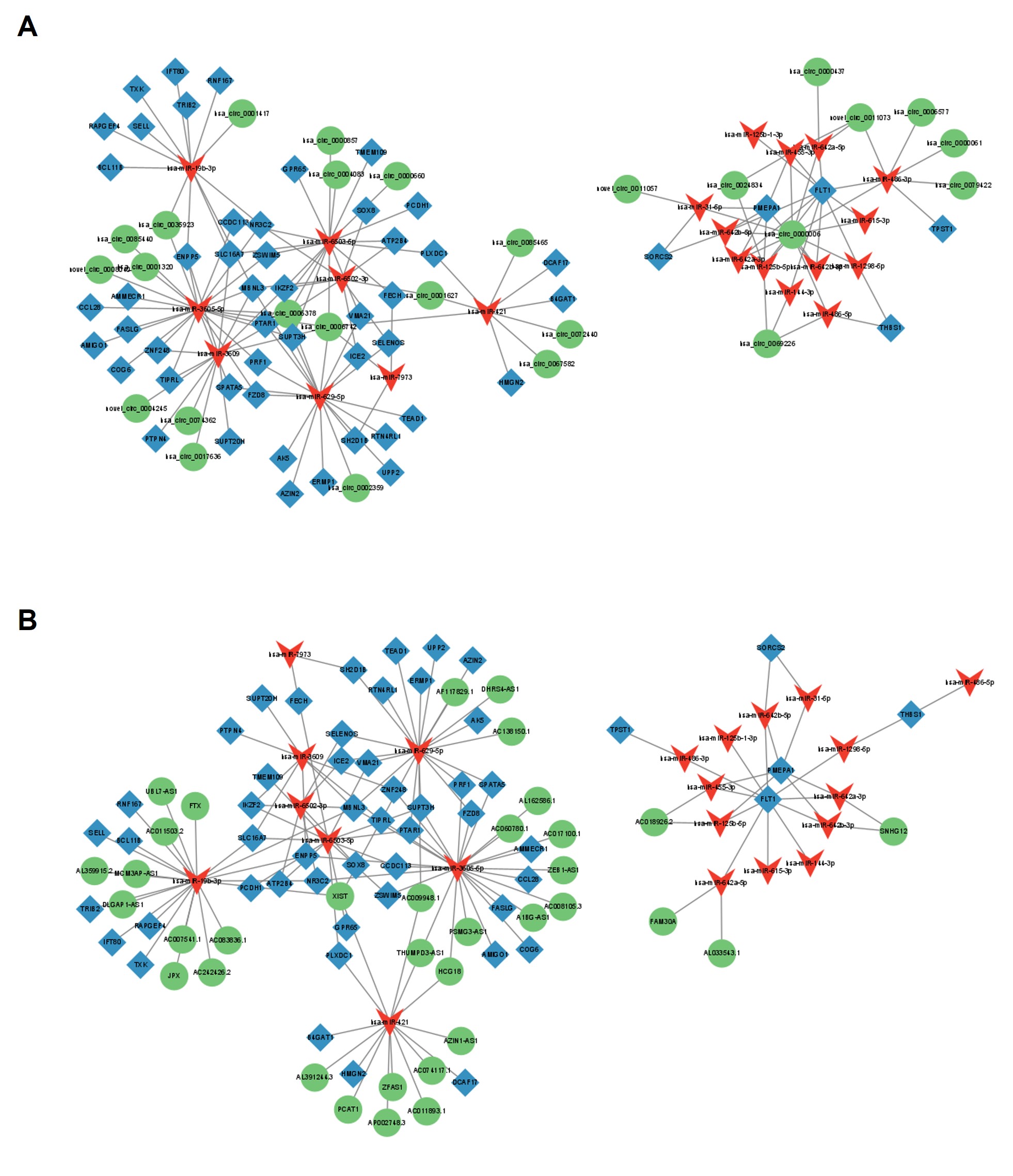

Supplement: Supplementary Figure S2 — The ceRNA networks in 140 overlapped genes. (A) The circRNA-miRNA-gene networks; (B) The lncRNA-miRNA-gene networks; [file Image2.jpeg]

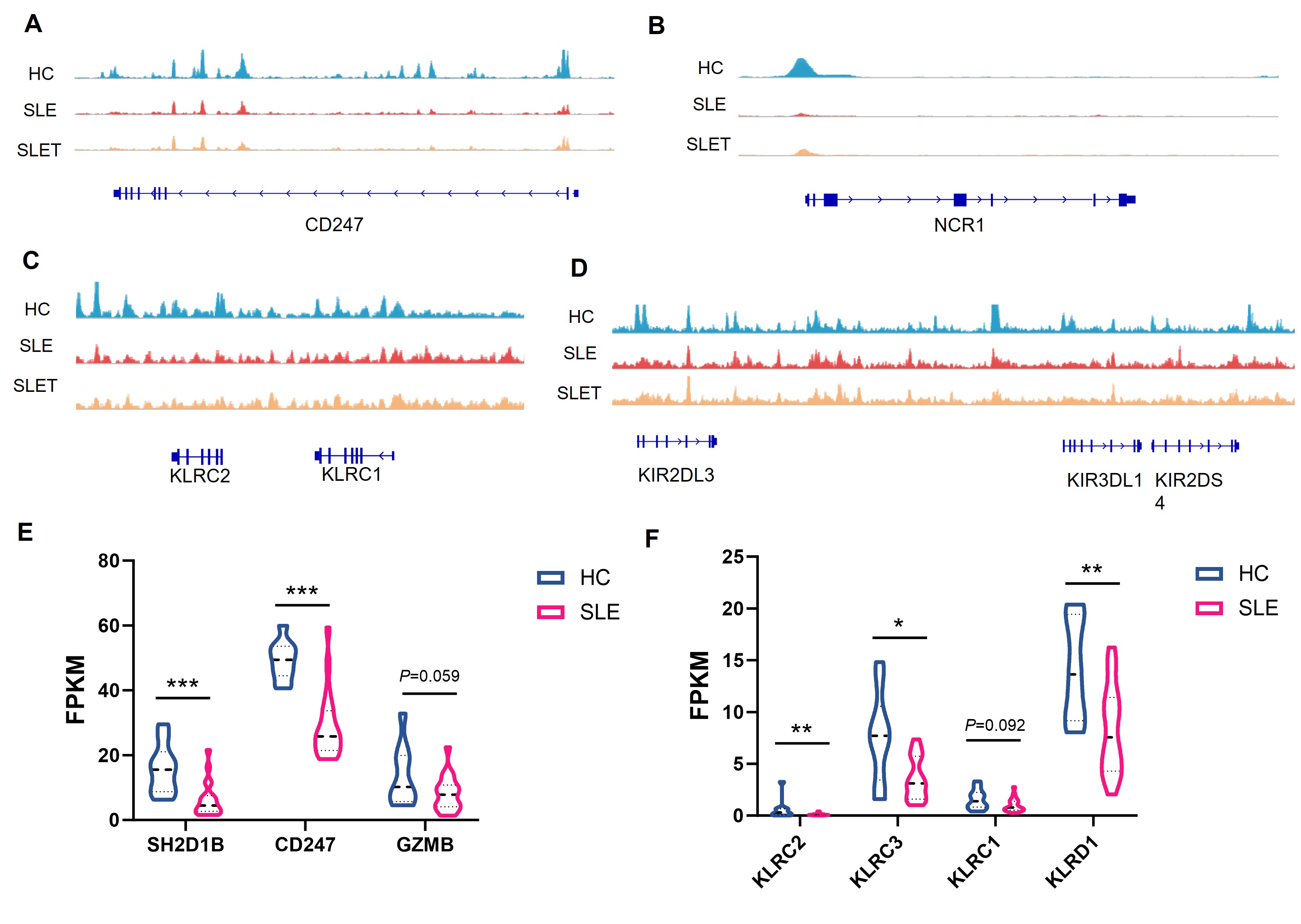

Supplement: Supplementary Figure S3 — Genomic localization and RNA-seq expression analysis of NK cell receptor genes. (A) IGV shows peaks located around NK cell receptor genes in different group; (B) The differential analysis of expression abundance for NK cell receptor genes in RNA-seq data from the public GEO database. ***P ≤ 0.001, **P ≤ 0.01, *P ≤ 0.05. [file Image3.jpeg]

**Supplementary Table S4:** **Transcription factor binding motifs enriched in DARs.**

**
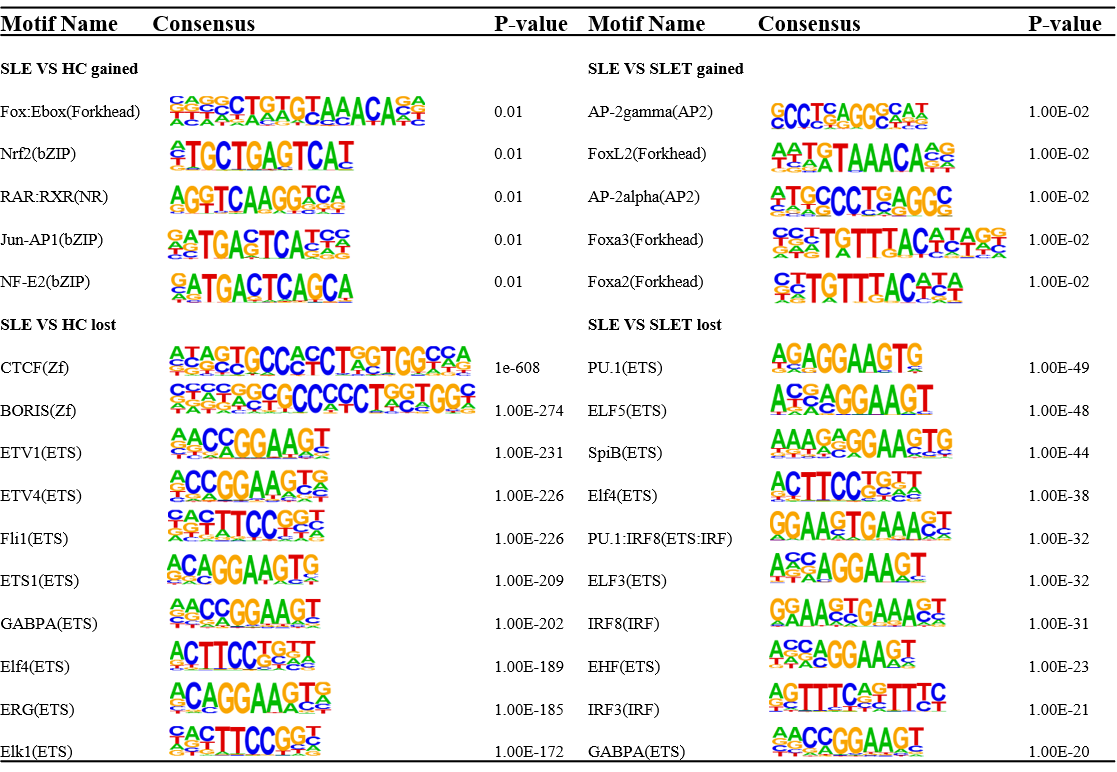
**

Supplement: Supplementary file 7 [file Table4.doc]
