# Supplementary material for: Multi-omics integration identifies NK cell-mediated cytotoxicity as a therapeutic target in systemic lupus erythematosus
Source: Front Immunol. 2025 May 13;16:1580540. doi: 10.3389/fimmu.2025.1580540 (PMC12106370; doi:10.3389/fimmu.2025.1580540)
Supplement: Supplementary file 4 [file Table1.doc]

**Supplementary TableS1:** Demographic and clinical characteristics of enrolled patients

|  | **HC** | **SLE** | **SLE after treatment** | **RA** |
| --- | --- | --- | --- | --- |
| **Demographic information** |  |  |  |  |
| No. of subjects | 7 | 6 | 6 | 7 |
| Age, years | 26(24,30) | 28.50(24.5,40.5) | —— | 57(51,62) |
| Sex(F/M） | 3/4 | 3/3 | —— | 6/1 |
| **Clinical andandcharacteristics** |  |  |  |  |
| SLEDAI | —— | 22.50(16,32) | 14.50(8,22) | —— |
| DAS28 | —— | —— | —— | 6.43(5.57,6.51) |
| ESR, mm/1h | —— | 31(16.75,44.50) | 18(13.50,45) | 46(12,63) |
| Low complement, % | —— | 100.00% | 75.00% | 33% |
| Anti-dsDNA, % | —— | 66.67% | 33.30% | —— |
| **Treatment** |  |  |  |  |
| Glucocorticoids, mg/d | —— | 25(18.13,57.50) | 11.25(8.75,21.25) | 8(5,8) |
| HCQ, % | —— | 100% | 66.67% | 42.86% |
| CTX, % | —— | 16.67% | 16.67% | —— |
| MMF, % | —— | 16.67% | 50% | —— |
| LEF, % | —— | 33.33% | 0% | 71.42% |
| TAC, % | —— | 16.67% | 50% | —— |

HC, healthy control; SLE, systemic lupus erythematosus; RA, rheumatoid arthritis; CRP, c-reactive protein; SLEDAI, Systemic Lupus Erythematosus Disease Activity Index; DAS28, disease activity score 28; ESR, erythrocyte sedimentation rate; Anti-dsDNA, anti-double-­stranded DNA; HCQ, hydroxychloroquine; CTX, cyclophosphamide; MMF, mycophenolate mofetil; LEF, leflunomide; TAC, tacrolimus.

Data were shown as median (interquartile range, IQR).
