# Supplementary material for: Multi-omics integration identifies NK cell-mediated cytotoxicity as a therapeutic target in systemic lupus erythematosus
Source: Front Immunol. 2025 May 13;16:1580540. doi: 10.3389/fimmu.2025.1580540 (PMC12106370; doi:10.3389/fimmu.2025.1580540)
Supplement: Supplementary file 5 [file Table2.doc]

**Supplementary Table S2: Demographic and clinical characteristics of validation cohort**

|  | HC | SLE |
| --- | --- | --- |
| **Demographic information** |  |  |
| Age, years | 34(27.25,49.5) | 36(27,46.5) |
| Sex(F/M） | 16/0 | 15/1 |
| **Clinical characteristics** |  |  |
| SLEDAI | —— | 11.50(8.00,19.00) |
| ESR, mm/1h | —— | 24.5(18.50,43.75) |
| Low complement, % | —— | 56.25% |
| Anti-dsDNA, % | —— | 53.33% |

HC, healthy control; SLE, systemic lupus erythematosus; SLEDAI, Systemic Lupus Erythematosus Disease Activity Index; ESR, erythrocyte sedimentation rate; Anti-dsDNA, anti-double-­stranded DNA.

Data were shown as median (interquartile range, IQR).
