# Supplementary material for: Multi-omics integration identifies NK cell-mediated cytotoxicity as a therapeutic target in systemic lupus erythematosus
Source: Front Immunol. 2025 May 13;16:1580540. doi: 10.3389/fimmu.2025.1580540 (PMC12106370; doi:10.3389/fimmu.2025.1580540)
Supplement: Supplementary file 6 [file Table3.doc]

**Supplementary Table S3: Primers designed for human mRNA detection**

| **Gene** | **Forward primer** | **Reverse primer** |
| --- | --- | --- |
| GAPDH | GCACCGTCAAGGCTGAGAAC | TGGTGAAGACGCCAGTGGA |
| SH2D1B | AGACAGCGAGTCGATACCAG | CCGTGTTTCTCTCTGAAGATTCG |
| CD247 | GGCACAGTTGCCGATTACAGA | CTGCTGAACTTCACTCTCAGG |
| GZMB | CCCTGGGAAAACACTCACACA | GCACAACTCAATGGTACTGTCG |
| KLRC2 | GCCAGCATTTTACCTTCCTCA | CACTGGGCTGATTTAAGTCGAT |
| KLRC3 | GCCAGCATTTTACCTTCCTCA | ATTGCACAGTTACGTTCAGCA |
| KLRC1 | AGCTCCATTTTAGCAACTGAACA | CAACTATCGTTACCACAGAGGC |
| KLRD1 | CAGGACCCAACATAGAACTCCA | GGAAATGAAGTAACAGTTGCACC |
